# Preservation of the External Jugular Vein in Bilateral Radical Neck Dissections: Technique in Two Cases and Review of the Literature

**DOI:** 10.1155/2015/168474

**Published:** 2015-01-06

**Authors:** Rodrigo Lima Bastos da Rocha, André Del Negro, Alfio José Tincani, Maíra Soliani Del Negro, Antonio Santos Martins

**Affiliations:** ^1^General Surgery Service, Department of Surgery, Faculty of Medical Sciences, State University of Campinas (UNICAMP), 13083-970 Campinas, SP, Brazil; ^2^Head and Neck Service, Department of Surgery, Faculty of Medical Sciences, State University of Campinas (UNICAMP), 13083-970 Campinas, SP, Brazil; ^3^Anesthesiology Service, Department of Anesthesiology, Faculty of Medical Sciences, State University of Campinas (UNICAMP), 13083-970 Campinas, SP, Brazil

## Abstract

*Context*. The possibility of cephalic venous hypertension with the resultant facial edema and elevated cerebrospinal fluid pressure continues to challenge head and neck surgeons who perform bilateral radical neck dissections during simultaneous or staged procedures. *Case Report*. The staged procedure in patients who require bilateral neck dissections allows collateral venous drainage to develop, mainly through the internal and external vertebral plexuses, thereby minimizing the risks of deleterious consequences. Nevertheless, this procedure has disadvantages, such as a delay in definitive therapy, the need for a second hospitalization and anesthesia, and the risk of cutting lymphatic vessels and spreading viable cancer cells. In this paper, we discuss the rationale and feasibility of preserving the external jugular vein. Considering the limited number of similar reports in the literature, two cases in which this procedure was accomplished are described. The relevant anatomy and technique are reviewed and the patients' outcomes are discussed. *Conclusion*. Preservation of the EJV during bilateral neck dissections is technically feasible, fast, and safe, with clinically and radiologically demonstrated patency.

## 1. Introduction

Since 1906, when Crile first described the radical neck dissection [1], this surgical procedure has been widely and systematically used to treat cervical metastasis in head and neck cancer. In most cases, this procedure is done unilaterally because of concern about the potentially hazardous consequences of simultaneous bilateral dissections. Simultaneous surgery is widely criticized because the ligature of both internal jugular veins (IJV) interrupts the major routes of venous blood return from the central nervous system (CNS). This may cause mild to severe facial edema, papillary edema with blindness, intracranial hypertension, coma, and, sometimes, death.

By 1950, with the availability of modern anesthetic techniques and routine tracheotomy, the staged procedure was accepted as feasible and relatively safe. Staged surgery allows the development of the internal and external vertebral venous plexuses and collaterals (emissary veins connecting the intra- and extracranial drainage systems). Other alternative routes include the deep cervical and the pharyngeal plexuses.

Comerota et al. [2] proposed that the internal jugular vein (IJV) could be reconstructed using either a spiral saphenous vein graft (a technique popularized by Doty in 1976) or polytetrafluoroethylene (PTFE) grafts. Although this approach yields excellent results, many disagree with this time-consuming technique, partly because it is prone to clot formation and thrombosis [3].

The preservation of the external jugular vein (EJV) was first proposed by Leclerc and Roy in 1932 [4] and later supported by others [3]. In a prospective study of Chung et al. it was showed that EJV preservation reduces immediate postoperative neck edema and pain or discomfort [5]. This easy-to-perform surgical maneuver was used here in two patients during bilateral simultaneous neck dissection, with no subsequent complications related to increased facial edema or CNS venous pressure.

The EJV is formed by the union of the retromandibular and posterior auricular veins, with the former vessel deriving from the confluence of the internal maxillary vein (directly from the pterygoid plexus that drains the cavernous sinus) and the superficial temporal vein. The EJV courses down and crosses the externocleidomastoid muscle obliquely, just under the platysma muscle on the superficial layer of the deep cervical fascia, before penetrating the fascia and entering the subclavian vein (in 70% of cases) or the IJV (in 30% of cases) [6].

The EJV is easily identified during physical examination, especially in thin patients. The depth of the skin incision over the vein during dissection of the neck requires special attention in order to preserve the vessel. Once identified under the platysma, the vessel can be spared by elevating the skin flaps along the plane of the vein. The EJV is approached during the superior flap elevation in the vicinity of the parotid gland and then preserving it after the dissection and ligature of all its collateral veins down to its junction with the ipsilateral subclavian vein.

## 2. Case Reports

### 2.1. Case #1

A 54-year-old male patient presented with a 5 cm wide lesion in the anterior floor of the mouth (FOM) that had persisted for seven months. Physical examination revealed invasion of the ventral portion of the tongue and alveolar ridge and a 3 cm node in the right submandibular triangle; the node was staged as T4aN1M0 squamous cell carcinoma (SCC). The patient underwent a right radical neck dissection, a left selective neck dissection (levels I, II, and III), marginal mandibulectomy, tumor resection, a pectoralis major myocutaneous flap, and tracheotomy.

**Figure 1:**
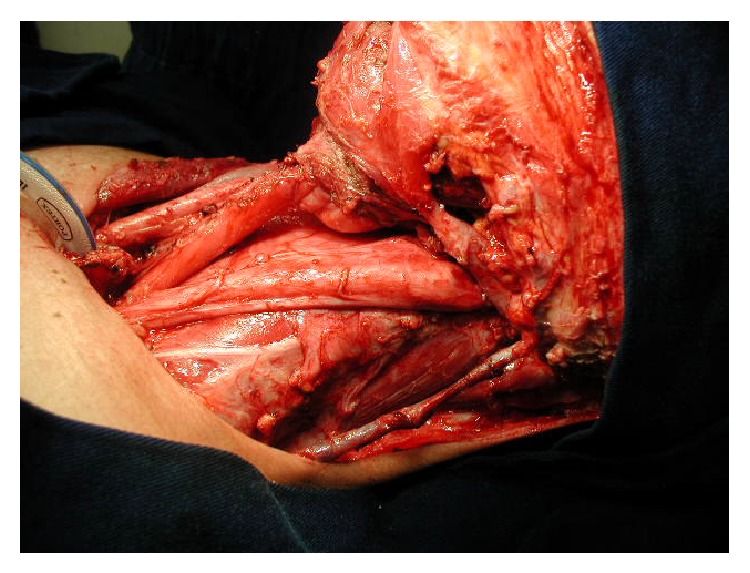
Left radical neck dissection with preservation of the left external jugular vein (EJV).

The pathologist's report indicated a grade II SCC, a microscopic positive surgical margin, no metastasis in 97 nodes examined, and one node with paracoccidioidomycosis. Postoperative radiotherapy was delayed because of local complications (mandible exposure and fistula). A needle biopsy done in the third month of follow-up revealed a 1 cm level I metastasis on the left side of the neck. The patient underwent a left radical neck dissection, with preservation of the EJV (Figure 1). This time, the pathologist's report showed three positive nodes (with extracapsular invasion) out of 21 examined.

**Figure 2:**
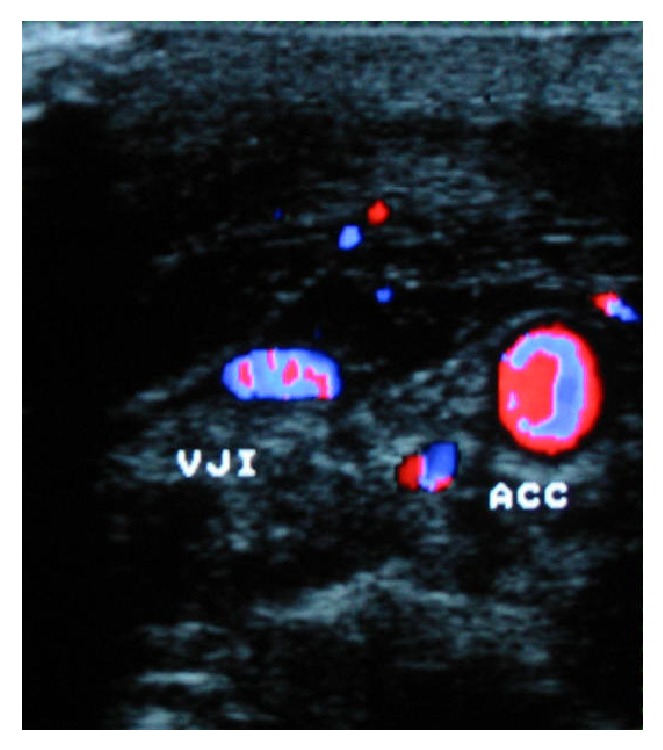
Postoperative duplex scan of case #1 showing patent left external jugular vein (EJV), collateral veins (C), and common carotid artery (CCA).

The patient received full postoperative radiotherapy at the primary tumor site and in the neck and did not receive chemotherapy because he developed kidney failure, and with such condition that kind of treatment was prohibitive. In the postoperative period, he developed mild facial edema but no symptoms of increased CNS venous pressure. An imaging scan with Doppler ultra-sound (duplex) in the early postoperative period and at 18 months of follow-up showed a patent EJV and the development of collateral veins (Figure 2). He lost follow-up 26 months after initial surgery without evidence of disease.

### 2.2. Case #2

**Figure 3:**
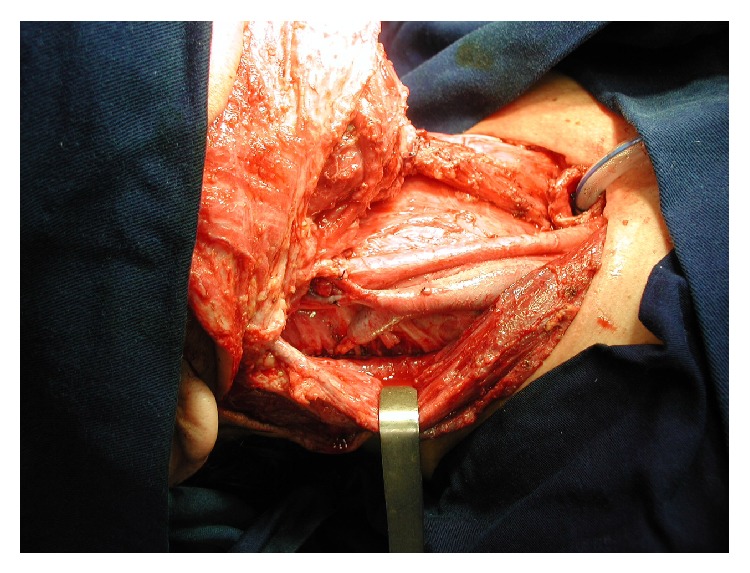
Right modified neck dissection (preserving the right thyrolinguofacial trunk and the right IJV distal stump).

A 43-year-old male patient presented with a tracheotomy tube inserted in Japan because of severe airway obstruction. Investigation revealed an extensive transglottic SCC, with invasion of the base of the tongue, left pyriform sinus, thyroid/cricoid cartilages, and strap muscles, as well as bilateral, clinically positive neck node metastasis (staged as T4aN2cM0). The patient underwent a total laryngectomy, partial pharyngectomy, left radical neck dissection (preserving the left EJV), and a right modified neck dissection (preserving the right thyrolinguofacial trunk and the right IJV distal stump) (Figure 3).

The pathology report showed a grade III transglottic SCC, three positive nodes out of 33 in the right neck, and five positive nodes out of 12 in the left neck, all with extracapsular invasion. Although the patient developed a pharyngocutaneous fistula, he did not experience any facial edema or changes in his mental status in the postoperative period. An early postoperative duplex scan showed a patent left EJV and a distal flux in the right IJV stump, below the level of the thyrolinguofacial trunk. Three months after surgery, the patient developed bone pain, and a bone scan revealed metastasis in the ribs and right proximal femur. The patient eventually developed retroperitoneal disease with bilateral pleural effusions and died five months after the initial treatment.

## 3. Discussion

The first radical neck dissection was done by Crile [1] and, in his original description, there was only one case of bilateral resection of the IJV as a staged surgery. Bilateral simultaneous resection of the IJV during neck dissection was considered to be a hazardous procedure because of the rapid elevation in intracranial pressure. Although reports of bilateral neck dissections began to appear in the 1950s [7], by 1970 there were several studies indicating a significant increase in the morbidity and mortality associated with bilateral radical neck dissections.

Moore and Frazell reported complications in 60% of their cases [8], with the most common being facial edema, sometimes of alarming extent. Other complications, such as fistulae, infection with wound dehiscence, and flap necrosis, were also common. In this same study, the postoperative mortality rate was as high as 14%, mainly because of CNS complications. More recent studies [9, 10] have also indicated high complication rates, but simultaneous bilateral neck dissections were done to preserve the contralateral IJV, even in patients with clinically palpable nodes. In these patients, adjuvant radiotherapy was always used for better local control.

The effects of a surgical intervention such as that described here are difficult to predict in a given patient. Although there are several ways of measuring intracranial pressure [11], many methods have been introduced to minimize or avoid the elevated intracranial venous pressure associated with bilateral neck dissections. These include staged neck dissections, modified neck dissections, internal vein reconstruction, and external jugular vein preservation. All these methods have their disadvantages. Thus, during staged neck dissections, which is the common procedure, there is a delay four to six weeks in the definitive treatment between the surgeries and when the EJV is preserved that leads to a reduction of 4 to 6 weeks of delay period and to a reduction of the complications of bilateral IJV removal also. Modified neck dissection with preservation of the IJV may be oncologically unsound in the presence of advanced neck metastasis, and reconstruction of the IJV with an autologous vein [2] or other graft is time-consuming and prone to thrombosis [3].

A review of the literature in the last 65 years revealed that few reports have dealt with IVJ reconstruction [2, 7] and elevated CNS pressure in neck dissections [11, 12]. The limited number of reports on EJV preservation in bilateral neck dissections [3, 4, 13] confirms the relevance of the present report. All previous papers are very old, except for an experimental study in rhesus monkeys [13] and a Chinese study that compares the conventional radical neck dissection, with dissection of the internal jugular vein, greater auricular nerve, and accessory nerve with a new technique, developed by them, named novel radical neck dissection. That is a regular radical neck dissection with preservation of the external jugular vein, greater auricular nerve, and deep branches of the cervical nerve. In this study, after the comparison of the two techniques, a statistically significant difference was revealed (*P* < 0.05) with concerning decrease of symptoms such as headache and dizziness due to preservation of the external jugular vein, as well as the reduction of the risk of intracranial hypertension due to preservation of the external jugular vein [14]. In the present investigation, both patients had uneventful outcomes, with minimal or no facial edema and no CNS complications. Duplex scans showed EJV patency in the early (both patients) and late (first patient) postoperative periods, as well as the development of collateral veins, thus confirming the validity of this procedure.

## 4. Conclusion

Preservation of the EJV during bilateral neck dissections is technically feasible, fast, and safe, with clinically and radiologically demonstrated patency. Since this procedure has a favorable postoperative outcome, preservation of the EJV is valid for patients submitted to simultaneous or staged bilateral neck dissections for metastatic disease. This procedure merits further investigation to determine whether there is a substantial reduction in the postoperative frequency of facial edema and high CNS pressure.
